# Telehealth by Home Monitoring and Video Consultation for Children With Cystic Fibrosis: Qualitative and Quantitative Study

**DOI:** 10.2196/80722

**Published:** 2026-05-26

**Authors:** Maria Nøregård Jørgensen, Kim Gjerum Nielsen, Mathias Aalling, Ragnheidur Traustadottir, Helena Hansson

**Affiliations:** 1 Paediatric Pulmonary Service, Department of Paediatrics and Adolescent Medicine, Copenhagen University Hospital, Blegdamsvej 9, 2100 København Ø, Copenhagen, 2200, Denmark, 45 35459875 ext 0045; 2 Department of Clinical Medicine, University of Copenhagen, Copenhagen, Denmark; 3 The Regional Capital, Centre for Health, Copenhagen, Denmark; 4 Department of Improvement and Digitalization, Copenhagen University Hospital, Copenhagen, Denmark

**Keywords:** telehealth, home monitoring, video consultation, feasibility, cystic fibrosis, children

## Abstract

**Background:**

Clinical outcomes for patients with cystic fibrosis have improved over the last decades, with a focus on enhancing the quality of life for children and their families. These improvements are expected to reduce the need for in-hospital visits, allowing for alternative care provisions that further enhance their quality of life. Telehealth can decrease the number of in-hospital visits and disruptions to everyday life.

**Objective:**

This study aims to assess the feasibility of home monitoring with subsequent video consultations and to explore the experiences of families and health care professionals.

**Methods:**

We recruited 12 children with cystic fibrosis (>6 y) and their parents from the Cystic Fibrosis-Center Copenhagen. The 5-month intervention consisted of alternately standard care visits and telehealth care visits. Telehealth care visits consisted of home monitoring, with automatically transmitted data to the hospital-based electronic health record. Home monitoring included spirometry, weight, and oxygen saturation, conducted with parental support, along with a video consultation. To monitor lung infections during telehealth care visits, all participants were encouraged to send in a sputum sample beforehand. Families completed preintervention and postintervention questionnaires about time spent, sense of security, and usability of home monitoring devices. We also collected data on families’ and health care professionals’ experiences with telehealth care visits through semistructured interviews after the intervention.

**Results:**

All 12 (100%) approached children and their parents accepted to participate. Standard care visits caused full-day absences for 8 (73%) children and 10 (91%) parents, compared to only 1 (8%) child and their parents during telehealth visits. More than 70% of children and their parents rated the oxygen saturation device (n=9, 75%) and weighing scale (n=11, 92%) as “very easy” to use after the intervention. However, 50% (n=6) found the home spirometry device “difficult” or “acceptable” to use, possibly due to device errors. We identified 4 main themes in the interviews: benefits of telehealth, disadvantages of telehealth, prerequisites for telehealth to be successful, and next steps toward telehealth.

**Conclusions:**

Home monitoring with automatically transmitted data to the hospital-based electronic health record and video consultations was feasible despite identified concerns regarding technical issues with home spirometry devices.

## Introduction

Cystic fibrosis is a chronic genetic disorder that primarily affects the lungs. It is caused by a mutation in the cystic fibrosis transmembrane conductance regulator (*CFTR*) gene, resulting in the production of thick, sticky mucus that obstructs the airways and increases the risk of lung infections [1]. Globally, the prevalence of cystic fibrosis is about 100,000 people [2,3]. Historically, cystic fibrosis has been associated with high mortality rates during childhood and adolescence, frequent hospital admissions, and reduced quality of life [4]. However, between 2006 and 2021, the median survival age increased significantly from 36.3 to 53.1 years [3]. Over the past few decades, clinical outcomes for both adults [5] and children from 6 years of age [6] with cystic fibrosis have progressively improved. These patients demonstrate improved lung function and health outcomes, along with a life expectancy nearly equivalent to that of the general population [5]. This improvement is largely attributed to the advent of the latest triple modulator therapy, a 3-drug combination that targets the underlying defect in the CFTR protein. Despite the improved health outcomes, patients with cystic fibrosis still require antibiotic treatments and continuous monitoring.

Patients with cystic fibrosis frequently attend in-hospital consultations with a physician, during which respiratory secretion samples are collected for microbiological analysis by a nurse, and pulmonary function is assessed via spirometry performed by trained pulmonary function technicians. The frequency of these visits varies across cystic fibrosis centers; however, best practice guidelines issued by the European Respiratory Society in 2018, prior to the introduction of new treatment, and in 2024, following its implementation, recommend conducting clinical assessments and respiratory secretion microbiology at least every 3 months [7,8]. In the United States, the 2024 clinical guidelines recommend conducting assessments every 4 to 6 months for children older than 6 years, rather than every 3 months as previously advised [9].

These frequent in-hospital visits are time- and resource-intensive for children with cystic fibrosis and their parents, often disrupting their everyday life [10]. Telehealth has the potential to reduce the number of in-hospital outpatient visits and to minimize such disruptions. During the COVID-19 pandemic, the use of various telehealth models for the cystic fibrosis population increased, with studies demonstrating the feasibility of home monitoring using spirometry, video consultations, and cellphone consultations for both adults [11,12] and children [13-15]. Only a few studies explored the feasibility of both home monitoring and video consultations [11,16] and home monitoring and cellphone consultations [12] in cystic fibrosis. These studies did not integrate home monitoring data directly into the hospital-based electronic health record (EHR) but used external systems and manual data entry. Currently, it is possible to integrate home monitoring data directly into most hospital-based EHR systems [17], thereby eliminating the need for external systems and manual data entry [11]. There is a lack of studies on both home monitoring with automatic data transmission to the hospital-based EHR and video consultations targeting children with cystic fibrosis and their families.

Additionally, telehealth for children requires parents to accept additional caregiving responsibilities. Studies highlight the importance of parents feeling secure in managing the treatment and care of children with long-term illnesses at home [18,19]. Telehealth also impacts the work procedures of health care professionals at the hospital [12]. Therefore, it is crucial to explore the experiences of parents and health care professionals with telehealth interventions to facilitate the best possible implementation. To investigate this further, the aim of this study is to examine the feasibility and experiences with telehealth, including home monitoring with automatic data transmission to the hospital-based EHR and video consultation, among children with cystic fibrosis, their families, and health care professionals. The findings of this study can potentially be adapted and applied to other chronic lung diseases.

## Methods

### Study Design and Population

This pilot study is a one-group pretest-posttest study evaluated with qualitative and quantitative methods. The study was conducted at the Cystic Fibrosis-Center Copenhagen in Denmark, which cares for approximately 100 children and adolescents aged 0 to 18 years, including patients from the Faroe Islands and Greenland.

We used criterion-based sampling to recruit children and parents [20]. The inclusion criteria for children with cystic fibrosis included (1) living 10 km or more from the hospital; (2) stable medical condition evaluated by a pediatric pulmonologist, defined by FEV1 (forced expiratory volume in 1 second) more than 80%, absence of chronic infections, and treatment with the latest triple modulator therapy for at least 6 months; and (3) aged 6 years or above. The inclusion criteria for parents were (1) having a child with cystic fibrosis participating in the intervention and (2) speaking Danish. A parent was defined as the child’s primary caregiver, regardless of whether the child was biological or adopted. Twelve children and their parents were approached during one of their standard care visits. We included 5 health care professionals, and the inclusion criteria to participate were (1) being employed at the Cystic Fibrosis-Center Copenhagen and (2) having a profession as a nurse, physician, or pulmonary function technician.

At the Cystic Fibrosis-Center Copenhagen, children are scheduled for an in-hospital outpatient visit every month. These visits last about 1 hour and include spirometry supervised by a pulmonary function technician, laryngeal suction for microbiology assessment conducted by a nurse, and a consultation with a physician.

### Telehealth Intervention

**Figure 1.**
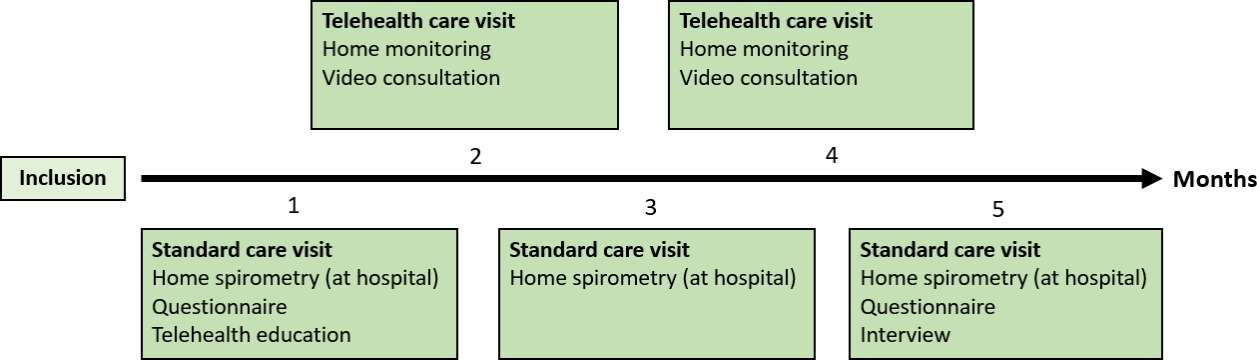
Intervention timeline over 5-month period for families and health care professionals. Standard care visits were conducted at the first, third, and fifth visits, and telehealth care visits were conducted at the second and fourth visits. The specific events at each visit are illustrated in the figure.

The intervention alternated between standard care visits and telehealth care visits over a 5-month period for each family (Figure 1).

#### Standard Care Visits

A standard care visit includes spirometry, microbiology assessment by laryngeal suction or sputum sample from the lower airways, and a consultation with a physician (Table 1). Standard care visits were conducted at the first, third, and fifth visits. At the first standard care visit, the families also received a suitcase with home monitoring devices and a smartphone if needed. The devices were connected to their own or a distributed smartphone, and families were trained to use the setup. Instructional materials for device functionality and video consultations were provided to minimize errors and facilitate the new practice. At the first, third, and fifth standard care visits, the child additionally performed 2 spirometry tests using their home spirometry device at the hospital. One test was conducted with support from a parent, while the other was conducted with support from a pulmonary function technician. The initial type of support was determined through randomization.

**Table 1.** Detailed description of procedures during standard care visits versus telehealth care visits.

Procedures of visit	Standard care visit	Telehealth care visit
Monitoring or measurements		
Weight and height	Weight and height measured before the lung function test	Weight measured
Oxygen saturation	Measured if FEV1^a^ <80% of the predicted value	Monitored
Spirometry	Measured with highly specialized stationary equipment allowing both inhalation and exhalation	Measured at home with a handheld device allowing only exhalation
Data collection	All measurements were recorded into the hospital-based EHR^b^ system manually	All measurements were automatically sent via Bluetooth (phone-internet connection) to the hospital-based EHR system (EPIC Systems Corporation) and were immediately available for health care professionals. Patients could access their health data securely through the web browser or mobile app (MyChart)
Respiratory secretion microbiology	Laryngeal suction or a sputum sample was obtained	It was recommended to send in a sputum sample. Participants from the Faroe Islands had access to laryngeal suctions at the Faroe Islands due to a temporary collaboration with the hospital in Torshavn
Consultations	The children and a parent had an in-hospital consultation with a pediatric pulmonologist, typically lasting about 30 minutes	Video consultations were facilitated via the hospital-based EHR system, enabling the pediatric pulmonologist to easily access participants’ home monitoring data before or during the video consultation

a FEV1: forced expiratory volume in 1 second.

b EHR: electronic health record.

#### Telehealth Care Visits

The telehealth care visit included monitoring of home spirometry, weight, and oxygen saturation, which were conducted at home by the children with support from a parent, along with a video consultation with a physician (Table 1). This process took place during the second and fourth visits (Figure 1). Participants from the Faroe Islands (n=4) had access to laryngeal suctions locally on the Faroe Islands during their telehealth care visits. Participants from Denmark (n=8) did not have any local agreements and were encouraged to send in a sputum sample. The hospital uses a hospital-based EHR system that allows home monitoring data to be transferred directly to the patient’s hospital record through a patient portal, accessible via a mobile app or computer. The portal automatically sends notifications 2 days before the scheduled video consultation to remind families to complete home monitoring timely.

### Monitoring Devices

The received suitcase contained a weighing scale from A&D Medical (model UC-450BLE), a wireless fingertip pulse oximeter from Nonin Medical (Elite 3240), and a handheld spirometry device from Spiromagic. All devices were equipped with Bluetooth capabilities and were compatible with our hospital-based EHR system (EPIC Hyperspace software, November 2024; EPIC Care). The devices were CE (Conformité Européenne) marked and validated. The handheld spirometry device used reference materials from the Global Lung Function Initiative, which were also utilized by the hospital spirometry equipment (MasterScreen, Jaeger, pneumotach). While the handheld spirometry device used the pitot method to measure flow, the hospital spirometry equipment used heated Lilly-type screen pneumotachographs. All devices were provided by the hospital.

### Quantitative and Qualitative Data Collection

Participant recruitment was carried out on a rolling basis during September and October 2022, followed by data collection from October 2022 through March 2023.

We assessed feasibility in terms of acceptability, demand, usability of home monitoring devices, sense of security, and impact on everyday life. Acceptability was evaluated by the number of participant agreements, while demand was assessed by the number of home monitoring measurements and video consultations from the hospital-based EHR system.

Questionnaires were completed on a tablet at the hospital during the first and the fifth standard care visits. The questionnaires included Likert scale and open-ended questions and were pretested for face validity with 5 families. Eleven families completed the preintervention questionnaire, and 12 families completed the postintervention questionnaire.

The usability of home monitoring devices was evaluated by questionnaires regarding expected and actual experience of operating home monitoring devices and comparing FEV1 data from the home spirometry device, as well as by semistructured interviews. We evaluated the sense of security and the impact on everyday life through questions about missing laryngeal suction and the amount of lost school and work hours during standard care visits and telehealth care visits. These aspects were also further assessed through interviews.

We conducted semistructured interviews with 6 families, each comprising 1 child and 1 parent, as well as focus group interviews with health care professionals. All interviews explored the families’ and health care professionals’ experiences with the telehealth intervention. Family interviews primarily relied on parents’ reflections, as obtaining independent perspectives from young children requires a substantially different and more extensive methodological approach (see Multimedia Appendix 1 for information on interview guide topics). Interviews were conducted by the co-author (MA), an experienced anthropologist, and lasted approximately 1 hour and were digitally recorded and transcribed.

### Data Analysis

We used descriptive statistics to analyze the demographic data and pre-post comparisons of questionnaire data of usability and everyday life. We analyzed interviews using thematic analysis as described by Braun and Clarke [21]. The transcribed data were read by MNJ and HH, during which initial coding and themes were elaborated. MNJ reviewed the dataset using a data-driven coding strategy to develop subthemes and main themes. Each theme was checked against coded extracts and the complete dataset to ensure meaningfulness. Finally, the relationships between the themes were discussed, rearranged, and renamed by MNJ and HH.

### Ethical Considerations

We applied to the Regional Research Ethics Committee for the Capital Region of Denmark. As the committee only reviews studies involving the collection of biological material, it determined that this study was not subject to its assessment (reference number F-22061333). The study was approved by the Danish Protection Agency (reference number P-2022‐694). In Denmark, all research studies must be approved by the Danish Protection Agency to ensure the safeguarding of patients’ sensitive personal data and compliance with the General Data Protection Regulation through appropriate technical and organizational measures. The study was conducted in accordance with the principles outlined in the World Medical Association Declaration of Helsinki.

The children, parents, and health care professionals received oral and written information about the study. Oral information was given by a pediatric pulmonologist and the project leader, both of whom have experience in recruiting children for research. The children and parents were assured that participation was voluntary and that they could withdraw from the study at any time without affecting the child’s treatment. They were assured of data confidentiality and the anonymity of the results. The children received age-appropriate information. Oral consent was obtained from all participants over 6 years of age, and written consent was obtained from participants over 14 years of age. Parents provided written consent on behalf of any child under 15 years of age. Oral information was provided to families by a pediatric pulmonologist and the project leader, both of whom have experience in recruiting children for research.

## Results

### Quantitative Questionnaires and Descriptive Data

Eight children lived in Denmark, while 4 lived on the Faroe Islands, resulting in a significant difference in the distance to the hospital (Table 2). All families agreed to participate in the study. Five health care professionals were approached: 3 physicians, 1 nurse, and 1 pulmonary function technician. All agreed to participate.

**Table 2.** Characteristics of the children with cystic fibrosis (n=12).

Characteristics	Values
Age (y), mean (SD)	11 (3.1)
Sex, n (%)	
Female	9 (75)
Male	3 (25)
Country, n (%)	
Denmark	8 (66)
The Faroe Islands	4 (33)
Distance to hospital (km), mean (SD)	
Danish participants	50.5 (37.5)
Faroese participants	1573 (6.2)
Using the latest triple modulator therapy, n (%)	12 (100)

#### Acceptability and Demand

**Figure 2.**
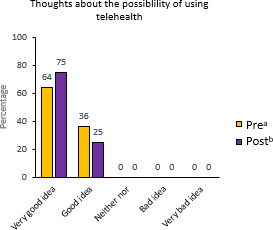
Responses to the questionnaire assessing the families’ thoughts about the possibility of using telehealth. ^a^In the preintervention questionnaire (n=11), we asked “What are your thoughts about the possibility of using telehealth?” ^b^In the postintervention questionnaire (n=12), we asked “What are your thoughts on continuing to have access to telehealth care?”

All 12 (100%) families completed the intervention through the submission of home monitoring data and participation in scheduled video consultations. Eleven (92%) families also completed both preintervention and postintervention questionnaires. Of the 6 families scheduled for postintervention interviews, all 6 (100%) participated, and the interviews were successfully conducted. These results indicate a strong willingness and interest to participate. All families wanted to continue having the possibility to use telehealth care visits, indicating a strong preference and demand for its implementation (Figure 2).

**Figure 3.**
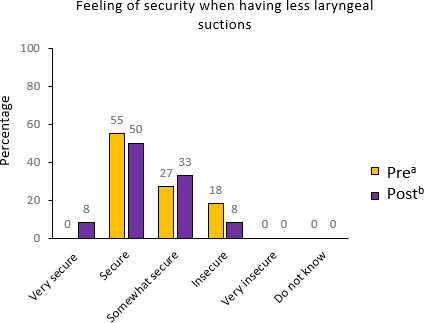
Responses to the questionnaire assessing the families’ sense of security with reduced laryngeal suction. ^a^In the preintervention questionnaire (n=11), we asked “How secure do you feel by having fewer laryngeal suctions during telehealth care visit?” ^b^In the postintervention questionnaire (n=12), we asked “How secure did you feel by having less tracheal suctions during telehealth care visit?”

Before the intervention, 2 (18%) of the 11 families reported feeling “insecure,” and none reported feeling “very secure” regarding the lack of laryngeal suction during telehealth care visits. After the intervention, the proportion of families feeling “insecure” decreased to 8% (1/12), while those feeling “very secure” increased to 8% (1/12). These findings indicate a shift toward greater feeling of security after the telehealth care visit (Figure 3).

#### Usability of Home Monitoring Devices

Of the 24 video consultations, 22 (91%) were successfully initiated, with only 1 consultation forgotten by a family and 1 encountering technical difficulties connecting to the hospital-based EHR system. All 12 families conducted home monitoring before the video consultation, though some data were delayed.

**Figure 4.**
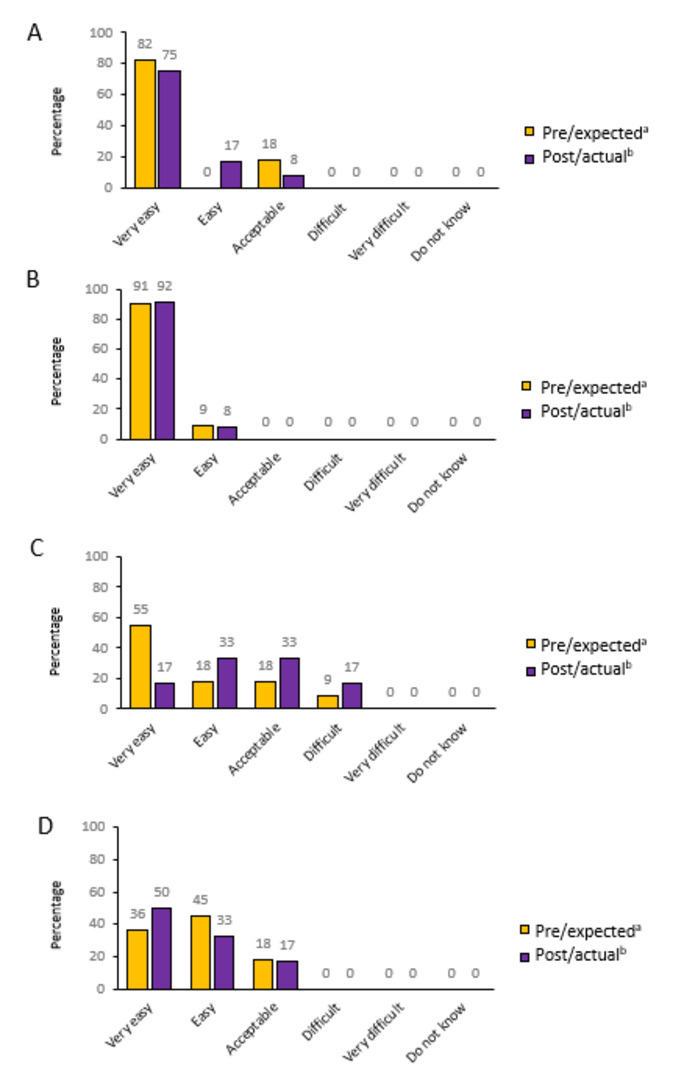
Questionnaire responses assessing families’ use of devices: (A) the oxygen saturation device, (B) the weighing scale device, (C) the home spirometry device, and (D) the video consultation. ^a^In the preintervention questionnaire (n=11), we asked “How do you think it would be operation X?” (pre/expected). ^b^In the postintervention questionnaire (n=12), we asked “How was it operating X?” (post/actual).

We encountered minimal difficulties with transferring data or connecting to home monitoring devices. Almost all families found the weighing scale and oxygen saturation monitoring to be “very easy” before and after the intervention (Figure 4A,B). Video consultations were rated slightly better post intervention (Figure 4C). However, the home spirometry device was rated more difficult to operate after the intervention (Figure 4D). FEV1 measurements from the home spirometry device were generally lower and more variable compared to those from the hospital spirometry equipment. Further examination revealed 2 significant software errors in the home spirometry device, which conflicted with Global Lung Function Initiative guidelines and hindered the validation of FEV1 measurements.

#### Impact on Everyday Life

**Figure 5.**
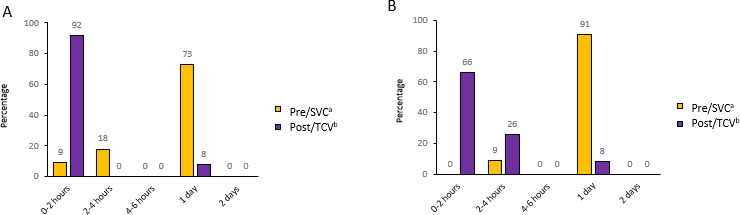
Questionnaire responses assessing missed school and time at work during a standard care visit versus telehealth care visit. (A) Missed school hours. (B) Missed work hours. ^a^In the preintervention questionnaire (n=11), we asked “How much time do you typically take off from school and work during a standard care visit?” ^b^In the post-intervention questionnaire (n=12), we asked “How much time did you take off from school and work during a telehealth care visit?”

Most families reported that they did not attend school or work during standard care visits (Figure 5): 73% (8/11) of the children and 91% (10/11) of their parents reported missing a full day of school or work due to standard care visit, whereas only 8% (1/12) of the children and their parents missed a full day when having a telehealth care visit. Additionally, 92% (11/12) children were absent for less than 2 hours during telehealth care visits. Families also reported time-saving benefits of telehealth, highlighting reduced transportation time.

### Qualitative Interviews With Families and Health Care Professionals

**Figure 6.**
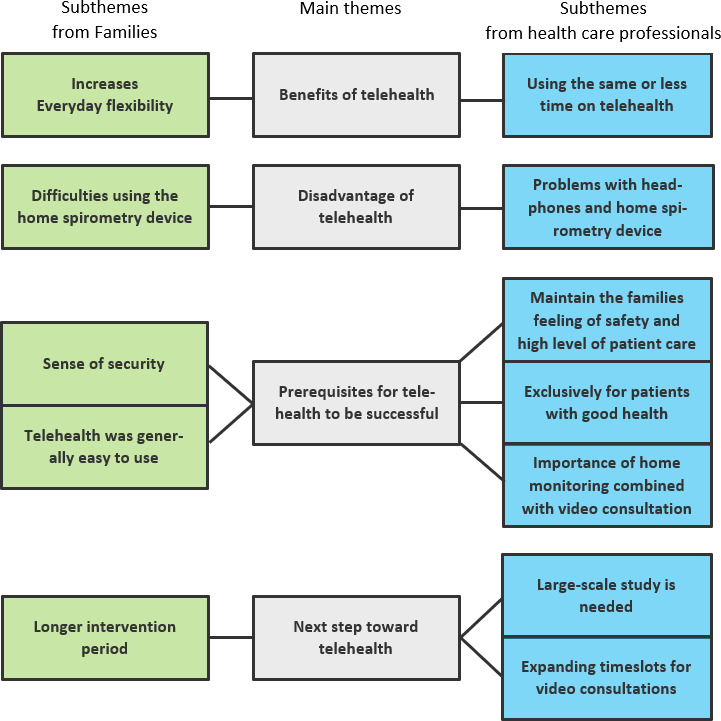
Main themes and subthemes from interviews with families and health care professionals. Subthemes from families are in green, and subthemes from health care professionals are in blue.

In the interviews, we examined the experiences of families and health care professionals with home monitoring and subsequent video consultations. We identified four main themes: (1) benefits of telehealth, (2) disadvantages of telehealth, (3) prerequisites for telehealth to be successful, and (4) next step toward telehealth. Furthermore, 12 subthemes were derived: 5 emerged from the family interviews and 7 from the interviews with health care professionals (Figure 6). An overview of the main themes and subthemes, including illustrative quotes, is presented in Table 3. In the following section, the main themes and subthemes are presented under the following sections: “Usability of Home Monitoring devices,” “Impact on Everyday Life,” and “Telehealth in the Future.”

**Table 3.** Main themes and subthemes with examples of quotes from interviews with families and health care professionals.

Main theme or subtheme	Quotes
Benefits of telehealth	
Increases everyday flexibility	“One thing is taking time off work ... but school ... that’s their future ... that’s where they have their friends and everything ...” (father to child 5, 9 years old).
Using the same or less time on telehealth	“... the [video consultation] doesn't take less time. Well, um ... the amount of small talk is perhaps a bit less ...” (health care professional). “There will be a task for the staff conducting lung function tests to train the families, but in return, there will be fewer lung function tests at the hospital” (health care professional).
Disadvantages of telehealth	
Difficulties using the home spirometry device	“The first time there were issues with the sound. It wasn’t on our end, but the doctor’s. There was no sound, so they had to call us on the phone” (father to child 2, 13 years old).
Problems with headphones and home spirometry device	“We had problems after we got digital recorders, because having both the headset and the digital recorder on the same computer caused issues. The system couldn’t figure out which microphone to use and things like that” (health care professionals).
Prerequisites for telehealth to be successful	
Easy to use telehealth setup	“I think the home monitoring has been fine. If it had been difficult or cumbersome, it would have been a challenge (...) And it has worked flawlessly” (father to child 1, 8 years old).
Sense of security	“We were told that we could just call if there was anything, and we can always come here. So, we haven’t felt insecure at any point at all” (father to child 1, 8 years old).
Exclusively for patients with good health	“The only thing I can say is that the Faroese patients are asking for it to be reinstated ...” (health care professional).
Maintain the families’ feeling of safety and high level of patient care	“If you see a lung function that has dropped by 20%, you just have to say, ‘You need to come in tomorrow’ ... So, in some way, I don't think people will feel insecure” (health care professional). “I also think it’s an important point that you say it’s here to stay to some extent ... so it makes sense and is still safe” (health care professional).
Importance of home monitoring combined with video consultation	“I think it’s extremely important to maintain the ability to measure something at home” (health care professional).
Next steps toward telehealth	
Longer intervention period	“Yes, and again, if it had run for a longer time, we might have come up with something more [a different perception]” (father to child 1, 8 years old).
Large-scale study is needed	“If it is to be scaled up, then other things might certainly emerge compared to this study, I think” (health care professional).
Expanding timeslots for video consultations	“But that’s something we could perhaps work with … then maybe some of those [early or late] times could be given” (health care professional).

#### Usability of Home Monitoring Devices

##### Main Theme: Disadvantage

###### Subtheme: “Difficulties Using the Home Spirometry Device”

Most families expressed uncertainty about the home spirometry device measurements. They noted difficulties in achieving lung function values comparable to those obtained with the hospital spirometry equipment and mentioned that the home spirometry device was hypersensitive to movement:

*That was the hardest [the home spirometry device] ... It’s not really something you can read about in a book because you have to instruct someone else on what to do.*

[Father to child 5, 9 years old]

Additionally, some families reported difficulties in hearing the physician during video consultations. Despite these audio issues, families did not find them very problematic, as physicians would call them by phone while maintaining video contact for visual communication.

###### Subtheme: “Problems With Headphones and Home Spirometry Device”

Health care professionals highlighted technical issues, including problems with the home spirometry device and frequent audio issues. One health care professional stressed that significant flaws in the home spirometry device software led to inaccurate lung function measurements. Health care professionals emphasized that telehealth must be easy and straightforward to be effective during a busy workday:

*Well, if it’s going to work, it should be like sitting down in the office or consultation room, and it should just work immediately. There shouldn’t be any fuss with it.*

[Health care professional]

### Main Theme: Prerequisites for Telehealth to Be Successful

#### Subtheme: “Easy to Use Telehealth Setup”

Families reported no issues connecting to the platform, and the video connection was satisfactory. Performing measurements was quick, and uploading the data was straightforward. Most families emphasized that user-friendly devices are essential for the success of telehealth.

#### Subtheme: “Sense of Security”

The parents described that the reduced frequency of standard care visits, and thereby reduced laryngeal suction, depended on their child’s current health. Some families emphasized the importance of having the option for an acute standard care visit if needed:

*We discussed that the prerequisite for reducing the frequency of physical visits is that the general well-being is good...*

[Father to child 5, 9 years old]

While all families felt secure during the intervention, they highlighted the need for a longer-term study to assess potential negative effects of telehealth.

#### Subthemes: “Maintain the Families’ Feeling of Safety and High Level of Patient Care” and “for Patients With Good Health”

The health care professionals highlighted that the intervention has been an excellent option for maintaining close contact to patients with cystic fibrosis and their parents and much better than, for example, “shared care.” They believe telehealth care visits primarily benefit children and their parents who frequently receive treatment and where health care professionals are familiar with the patient’s medical history. The health care professionals identified the Faroese patients and their parents as the group that benefits the most from telehealth care visits due to the distance to the hospital:

*But this is a solution model that maintains close contact ... A good solution for patients who know us.*

[Health care professional]

Health care professionals emphasized that families should have access to acute visits if home measurements were concerning or if they felt unsafe, enhancing their sense of security. Health care professionals noted that video consultations are superior to cellphone consultations as physicians can observe the child and notice symptoms, such as coughing. Most health care professionals agreed that telehealth will play a significant role in future health care and stressed the importance of a suitable strategy. Additionally, health care professionals collectively agreed that patient safety should be prioritized, given the evidence of low infection risks and high-quality home monitoring.

#### Subtheme: “Importance of Home Monitoring Combined With Video Consultation”

The health care professionals emphasized the importance of continuing to obtain objective measurements of patients’ condition. For the majority of individuals with cystic fibrosis, cellphone or video consultations alone are considered insufficient.

### Impact on Everyday Life

#### Main Theme: Benefits of Telehealth

##### Subtheme: “Increasing Everyday Flexibility”

According to the parents, hospital flexibility is essential for maintaining normalcy in their lives. Most families reported that telehealth significantly facilitated their daily routines by enabling assessments to be conducted at any time and location, which they regarded as a major advantage:

*It makes it much easier for us ... we can conduct measurements a few days in advance ... if we have plans for the weekend, we can actually go to the summer house.*

[Father child 1, 8 years old]

They also indicated that involvement in the telehealth intervention afforded them more time for everyday activities because it reduced the need to travel to and from the hospital. Parents expressed relief over increased school attendance and more opportunities for their children to build friendships.

##### Subtheme: “Expanding Timeslots for Video Consultations”

To further enhance telehealth benefits, families suggested scheduling video consultations either early in the morning or later in the afternoon, as midday consultations require parents and children to leave and return to work and school. Simultaneously, health care professionals described that families generally preferred later video consultations, necessitating adjustments in clinic schedules. A proposed schedule included standard care visits until noon and telehealth care visits later in the day, thereby reducing clinic traffic.

##### Subtheme: “Using the Same or Less Time on Telehealth”

Health care professionals expressed that the time spent on telehealth care visits compared to standard care visits varied by profession. Physicians spent a comparable amount of time on both video and standard consultations. However, video calls were sometimes completed more quickly due to reduced small talk. Without the need for in-hospital spirometry measurement and laryngeal suction at telehealth care visits, overall time savings were anticipated, particularly for nurses and pulmonary function technicians. However, the task of training families to use telehealth devices would be an added responsibility.

### Telehealth in the Future

#### Main Theme: Next Steps Toward Telehealth

##### Subthemes: “Longer Intervention Period” and “Large-Scale Study Is Needed”

Despite positive experiences with telehealth care among all families, some expressed a preference for an extended trial duration. They found it challenging to render a comprehensive evaluation of the intervention based on only 2 telehealth care visits. Several families suggested a 1-year intervention period with 6 telehealth care visits in total:

*A little more time in the experiment would have been beneficial. It would have allowed us to get everything properly in place.*

[Mother to child 6, 6 years old]

The health care professionals highlighted the necessity of a longer intervention period to provide more comprehensive feedback, as the pilot study had a small sample size and might fail to detect relevant aspects. Physicians expressed concerns about the absence of the microbiology testing during telehealth care visits, which could lead to undetected lung infections and worsen patient care. Therefore, they advocated for a large-scale study to ensure patient safety by collecting data on lung infections over a longer telehealth care period.

##### Subtheme: “Expanding Timeslots for Video Consultations”

Since families have expressed a preference for video consultation appointments later in the day, rather than at the current time slots between 9 AM and 2 PM, the health care professionals agreed that it is essential to review and potentially adjust clinic timeslots. Some health care professionals noted that clinic traffic would be significantly reduced at noon compared to the afternoon rush hour, which could facilitate scheduling changes. However, this change will need to be carefully considered and will not be an easy task:

*I definitely think that patients can save some time, especially if these appointments are scheduled at off-peak times … Because I don’t think it makes sense to have a video consultation at 11 or 12 or something like that.*

[Health care professional]

## Discussion

### Principal Results and Comparison With Prior Work

We examined the feasibility of telehealth with home monitoring, which involved automatic data transmission to the hospital-based EHR and video consultations, from the perspectives of families and health care professionals. Our findings indicated that home monitoring and video consultations were feasible. However, we identified concerns related to home spirometry.

In our study, all participating families expressed a strong willingness and interest in engaging in the telehealth intervention and desired to continue using it. This contrasts with research by Gur et al [22] who reported some difficulties recruiting children with cystic fibrosis older than 8 years of age to an intervention period with Skype-based video consultations and WhatsApp messages without home monitoring. Similarly, Compton et al [11] found that after implementing 100% telehealth care visits for adults with cystic fibrosis during COVID-19, 32% wished to revert to in-hospital standard care visits after 1 telehealth care visit. Only one-third of the participants had access to a home spirometry device. The willingness and interest to participate in our study could be attributed to the implementation of only 50% telehealth care visits and the ability for all participants to automatically transmit monitoring data (such as spirometry). Although our participants had access to relevant home monitoring devices, only one-third of participants in the study by Compton et al had, and none in the study by Gur et al. The presence of home monitoring in our study may have had a positive influence on participants’ willingness to continue and commit to telehealth care visits, compared to the findings of Compton et al and Gur et al. Additionally, Gifford et al [13] found that 61 out of 127 pediatric cystic fibrosis programs in the United States identified home monitoring as the most crucial enhancement needed to improve the rating of telehealth solutions for patients with cystic fibrosis. However, due to the urgency, many studies conducted during COVID-19 included only phone calls [23] or video consultations [22]. From the perspective of health care professionals in this study, video consultations are superior to phone calls, as they allow the physician to both see and hear the child. Additionally, home monitoring measurements are deemed essential for the treatment and follow-up of patients with cystic fibrosis during telehealth care visits. Ideally, physicians would prefer to receive respiratory secretion microbiology results at all telehealth care visits to minimize the risk of overlooking respiratory infections. Unfortunately, it is not yet possible to detect lung infections at home, although research in this area is advancing, for example, through the use of electronic nose technology to detect airway colonization through breath analysis [24].

In this study, both families and health care professionals highlighted the patient’s sense of security as an essential prerequisite for successful telehealth. While families in general felt secure, the lack of monthly laryngeal suction was the most concerning issue. This concern aligns with the findings from a multicenter study on patients with cystic fibrosis who transitioned to video or cellphone consultations without home monitoring during COVID-19. A survey conducted in the study revealed that 64% of parents of children with cystic fibrosis expressed at least moderate concern about the absence of respiratory secretion microbiology during the telehealth period [25].

Although the families in this study expressed concern about the absence of respiratory secretion microbiology, the level of concern was somewhat reduced after the intervention. This may be due to an experienced reduced risk of lung infections after the new treatment therapy. Future research could investigate educating patients to collect induced sputum samples at home as an alternative to laryngeal suction, despite the challenges many cystic fibrosis patients face in producing induced sputum after therapy treatment.

To enhance patients’ sense of security, the health care professionals emphasized the importance of clearly communicating that families could receive acute standard care visits if needed. They also highlighted that monitoring lung function and oxygen saturation enables early detection of exacerbations, providing physicians with crucial information for timely treatment and further reassurance. Additionally, for future intervention, artificial intelligence–assisted home stethoscope monitoring has proven to be reliable in detecting exacerbations and has the potential to offer valuable insights for early intervention and effective management [26]. A review study further demonstrated that early and acute decline detection measured from home monitoring devices was associated with reduced acute care use [27]. These findings support our findings that health care professionals prioritize early exacerbation detection to improve patients’ sense of security.

Families in our study found video consultation and home monitoring convenient, saving them time compared to standard care visits. Most families increased their time spent at school and work from 2 hours to 1 day when having telehealth care visits instead of standard care visits. This aligns with the study by Rodkjær et al [12], where adults with cystic fibrosis reported time savings and increased flexibility after transitioning from in-hospital visits to home monitoring of spirometry and cellphone consultations and an overview study [28]. However, most families in this study found it inconvenient that video consultations were scheduled in the middle of the day, as both the parent and child had to leave school and work to attend. Families suggested scheduling video consultations either early in the morning or later in the afternoon. The health care professionals are aware of the families’ preferences and suggest that the cystic fibrosis center may need to be restructured to accommodate families’ wishes.

The inclusion of 4 patients from the Faroe Islands highlighted a significant contrast in hospital distance compared to Danish patients. Despite the greater distance, 8 (73%) of the 11 children and 10 (91%) of the 11 parents missed an entire day of school and work during standard care visits, indicating that many Danish families also find it challenging to attend school and work during standard care visits even though the distance is relatively short. The difficulty of attending school or work may arise from standard care visits scheduled during midday, compounded by parental uncertainty regarding traffic conditions and hospital waiting times. These aspects underscore the potential value of implementing home monitoring and video consultations to reduce these burdens.

Both families and health care professionals emphasized that home monitoring and video consultations must be highly user-friendly to be preferred in the future. In this study, most monitoring devices were rated as easy to use, except for the home spirometry device. The families expressed no problems with the automatic transmission of data to the hospital-based EHR system and highlighted that home monitoring, in general, was easy and straightforward. In a realist review study by Thomas et al [27], it was identified that when using home monitoring, it was important to provide timely care to prevent hospital admission. To enhance timely care, automatically transferred data were preferred to reduce errors and delays due to manual typing. It is possible that the families’ and health care professionals’ concerns about the home spirometry device are attributable to the software errors identified. Given that the home spirometry device has the capability to automatically transmit data to our hospital-based EHR system, we decided to further develop the device before initiating a large-scale study and implementation.

### Strengths and Limitations

The strength of this study is its exploratory prospective design, which makes it possible to gain insights into both the families’ and health care professionals’ perceptions of a limited period of telehealth care. It provides valuable insights for refining and adjusting the intervention before a large-scale study and implementation.

The pilot study also had several limitations. Due to the small sample size of 12 families and 5 health care professionals and the absence of a control group, our results should be interpreted with caution and should be verified in a large-scale study including more telehealth care visits. Families were selected based on their stable medical condition and general health, with safety prioritized; this may limit the generalizability of the findings. The questionnaires used were pilot tested but not further validated. In a large-scale study, it would be valuable to use validated questionnaires due to reliability, validity, and comparability. Furthermore, software errors in the home spirometry device compromised data quality and prevented reliable comparison of FEV₁ values between home and hospital measurements. These errors also negatively affected user experience, meaning that the reported usability ratings reflect issues with the software rather than the device itself. Therefore, interpretations of device usability should be made with caution. Finally, as 2 of the authors were involved in delivering the intervention, there is a potential risk of observer and social desirability bias, which may have influenced participant responses and the interpretation of the findings. Nevertheless, all interviews were conducted by an author who was not involved in the intervention and had no prior relationship with the participants.

### Conclusions

In summary, this pilot study demonstrates that telehealth incorporation—home monitoring with automated data transmission and video consultations with children and their parents—is feasible and highly acceptable. The study provides important knowledge about adjustments needed to be made in the telehealth setup before initiating a large-scale study and implementation in routine clinical practice. A large-scale study could be extended to other groups of patients with chronic lung diseases focusing on usability and safety in an adjusted telehealth care setup.

## Supplementary material

10.2196/80722 Multimedia Appendix 1 Interview guide topics.

## Abbreviations

CE: Conformité Européenne
EHR: electronic health record
FEV1: forced expiratory volume in 1 second
