# Supplementary material for: Telehealth by Home Monitoring and Video Consultation for Children With Cystic Fibrosis: Qualitative and Quantitative Study
Source: JMIR Form Res. 2026 May 26;10:e80722. doi: 10.2196/80722 (PMC13205462; doi:10.2196/80722)
Supplement: Multimedia Appendix 1 [file formative-v10-e80722-s001.docx]

**Supplementary 1**

**The interview guide for the families covered the following topics:**

**1) General telehealth**

**2) Video consultations**

**3) Home monitoring and MyChart**

**4) Telehealth and everyday life.**

**The interview guide for the HCPs included topics such as:**

**1) Experienced workflow during telehealth care visits**

**2) Clinical quality**

**3) Technology and equipment.**
